# Testing Dietary Hypotheses of East African Hominines Using Buccal Dental Microwear Data

**DOI:** 10.1371/journal.pone.0165447

**Published:** 2016-11-16

**Authors:** Laura Mónica Martínez, Ferran Estebaranz-Sánchez, Jordi Galbany, Alejandro Pérez-Pérez

**Affiliations:** 1 Secció de Zoologia i Antropologia, Departament de Biologia Evolutiva, Ecologia i Ciències Ambientals, Facultat de Biologia, Universitat de Barcelona, Barcelona, Spain; 2 Center for the Advanced Study of Human Paleobiology, Department of Anthropology, The George Washington University, Washington DC, United States of America; New York Institute of Technology, UNITED STATES

## Abstract

There is much debate on the dietary adaptations of the robust hominin lineages during the Pliocene-Pleistocene transition. It has been argued that the shift from C3 to C4 ecosystems in Africa was the main factor responsible for the robust dental and facial anatomical adaptations of *Paranthropus* taxa, which might be indicative of the consumption of fibrous, abrasive plant foods in open environments. However, occlusal dental microwear data fail to provide evidence of such dietary adaptations and are not consistent with isotopic evidence that supports greater C4 food intake for the robust clades than for the gracile australopithecines. We provide evidence from buccal dental microwear data that supports softer dietary habits than expected for *P*. *aethiopicus* and *P*. *boisei* based both on masticatory apomorphies and isotopic analyses. On one hand, striation densities on the buccal enamel surfaces of paranthropines teeth are low, resembling those of *H*. *habilis* and clearly differing from those observed on *H*. *ergaster*, which display higher scratch densities indicative of the consumption of a wide assortment of highly abrasive foodstuffs. Buccal dental microwear patterns are consistent with those previously described for occlusal enamel surfaces, suggesting that *Paranthropus* consumed much softer diets than previously presumed and thus calling into question a strict interpretation of isotopic evidence. On the other hand, the significantly high buccal scratch densities observed in the *H*. *ergaster* specimens are not consistent with a highly specialized, mostly carnivorous diet; instead, they support the consumption of a wide range of highly abrasive food items.

## Introduction

The emergence of *Paranthropus* and *Homo* lineages in East Africa has been linked to an ecological shift toward C4 grasslands between 2.4 and 1.8 million years ago (Ma) caused by a marked global cooling and drying that resulted in contrasted year-round seasons and a variety of ecological scenarios with great spatial heterogeneity and ecological instability [1–6]. Remains of both *Paranthropus boisei* and early *Homo* have been associated with both well-watered, riverine habitats with gallery forest and woodlands in older localities and with extensive dry grasslands with episodes of lake fluctuations or, more recently, deltaic conditions. This habitat shift is assumed to have forced hominines to adopt a more intense exploitation of savanna plant foods, including underground storage organs (USOs). The robust australopithecines would have relied on dental and facial morphological adaptations to cope with long-term environmental challenges, whereas the generalized use of lithic tools would have offered early *Homo* greater opportunities to exploit food resources in highly variable environments [7–10]. The massive mandibular corpus, extended muscle insertion areas on the skull, large occlusal molar surfaces, premolar molarization, and thick enamel layers in *Paranthropus* are consistent with consumption of hard foodstuffs involving strong cracking, crushing, and grinding activities [11–14]. Tooth chipping and massive occlusal wear on the postcanine dentition of *Paranthropus* are indicative of peak bite forces and frequent chewing of small, hard food abrasives [15,16]. However, occlusal dental microwear analyses of *Paranthropus boisei* teeth fail to reveal any evidence of hard object feeding and contrast with isotopic evidence supporting a diet based above 70% on C4 plants such as fibrous grasses, sedges, or rhizomes [17–20]. Many fallback foods are mechanically challenging, which may explain the high occlusal wear of *Paranthropus* teeth [21], while the reduced dental and facial proportions in early *Homo* have been interpreted as indicative of meat exploitation as a major food source, mainly through scavenging strategies [22–24] to offset the dearth of succulent food resources in open environments [25,26]. Numerous studies have emphasized the importance of meat consumption in the large brained, small-toothed hominines [27–29]. However, the reduction in tooth size would have potentially limited the types of foods available to *Homo ergaster*. In contrast, in *Homo habilis*, the dental reduction that characterizes more recent humans was not fully attained [30,31].

Dental microwear patterns, both on buccal and occlusal enamel surfaces, have proved to be highly informative of foraging strategies in extant primates [32–35]. Early analyses of occlusal microwear patterns on *A*. *africanus* and *P*. *robustus* suggested that "the diet *of Paranthropus* entailed the mastication of harder items than composed the dietary staples *of Australopithecus*", similar to those of primates that eat large quantities of hard objects [36,37]. In contrast, more recent analyses of occlusal enamel texture suggested that *P*. *boisei* might not have consumed extremely hard or tough foods in the days prior to death [18] and that it might have consumed foods “with similar ranges of toughness as those eaten by *A*. *africanus*” [19]. Early *Homo* would not have relied on extremely hard or tough foods such as nuts, USOs or dried meat, whereas *H*. *ergaster* would have consumed more fracture-resistant food items (USOs or tough animal tissues) than *H*. *habilis* [38]. The two *Homo* species would have differed in fallback food consumption during stress periods, consistent with the climate change towards open savannas over time, with *H*. *ergaster* relying on stone tools for processing fallback foods [39,40].

Dietary hypotheses based on occlusal dental microwear research can be tested by buccal dental microwear patterns analyses. Buccal dental microwear is characterized by numerous striations with varying orientations and the lack of other wear features [35,41–44], such as pits or inter-tooth attrition that are common on occlusal enamel surfaces. Occlusal microwear patterns vary between shearing and grinding facets on the same tooth [45] and intra-facet variability within molar teeth has been shown to depend on varying mastication processes [46]. Occlusal dental microwear is highly affected by dental gross wear, because dentine exposure and enamel cracking quickly wear away the Phase II molar facets upon which most occlusal microwear research is based. In addition, forceful tooth-to-tooth contact and dental grinding are non-dietary sources of microwear features (both pits and scratches) on occlusal surfaces [45,47]. In contrast, buccal microwear is not affected by occlusal wear and dentine exposure [48] and has been shown to have a clearly distinct *in vivo* feature formation dynamics, and is likely to have a longer formation span than occlusal surfaces [49–51]. Buccal microwear is the result of the interaction of abrasive particles, such as plant phytoliths or silica dust, with the buccal enamel surfaces of teeth during chewing [52,53], as food particles move around in the mouth (mainly in an up-to-down and front-to-back direction) until they are swallowed.

It has been shown that post-depositional, taphonomic processes do not add new microwear features; instead, they obliterate and erase them, significantly damaging enamel surfaces, as shown by experimental analyses [54,55], which makes *post-mortem* damage clearly distinguishable from *ante-mortem* diet-related microwear patterns [35,56,57]. The presence of pits on the buccal surfaces and of microwear features on inter-proximal wear facets is a clear indicator of *post-mortem* damage [35,58].

Buccal microwear patterns in humans have been shown to be age-dependent in archeological collections [43] and in Middle and Upper Pleistocene fossil specimens, especially in juvenile individuals with definitive, fully functional dentition [59]. However, the intra-population variability of buccal microwear patterns has been shown to be smaller than the inter-population variability in adult individuals of hunter-gatherer populations from different ecological areas [41,53]. Buccal microwear patterns analysis is a replicable procedure [60] and has been shown to be highly dependent on ecological constraints and dietary preferences in both extant and fossil primates [33] and in fossil hominins [41,43,61,62]. Consequently, buccal microwear research is informative of diet composition and on the amount of abrasives incorporated to foodstuffs during food processing [41,62–64].

In the present research, scratch densities and average lengths by orientation categories on well-preserved teeth of *P*. *boisei*, *H*. *habilis* (early *Homo*), and *H*. *ergaster* specimens are studied. Their buccal microwear patterns are compared to those of extant primate samples from both closed forests and open woodlands and to those of the previously studied hominins *Australopithecus anamensis* [65] and *Australopithecus afarensis* [66] specimens. The main goals are to test the contradictory interpretations derived from anatomical traits, occlusal microwear patterns and texture data, and isotopic evidence for the robust australopithecines from East Africa and to determine the significance of a carnivorous diet in the *Homo* clade.

## Materials and Methods

### Samples studied

A total of 446 postcanine teeth were analyzed (Table 1), belonging to 167 fossil specimens of *Paranthropus aethiopicus* (N = 44), *Paranthropus boisei* (N = 56), *Homo habilis* (early *Homo*) (N = 49) and *Homo ergaster* (N = 18) from East African sites dating from 2.5 to 1.4 Ma, including Omo and Hadar in Ethiopia, Koobi Fora, West Turkana, Lake Baringo, and Lainyamok in Kenya, and Olduvai Gorge and Peninj in Tanzania. All necessary permits were obtained for the described study from the Tanzania Commission for Science and Technology (COSTECH), the Kenyan National Commission for Science, Technology and Innovation (NACOSTI), and the Nairobi National Museum, which complied with all relevant regulations. The studied samples included the same specimens for which occlusal dental microwear and texture patterns have been previously studied [10,19,38,67,68]. Dental molds were made from the original fossil specimens curated at the National Museums of Ethiopia (Addis-Ababa), Kenya (Nairobi) and Tanzania (Dar es Salaam and Arusha). Taxonomic attributions of hominin specimens were obtained from the literature [69–75]. However, there is no full consensus concerning the taxonomic attribution of some early *Homo* specimens to *H*. *habilis*, *Homo rudolfensis*, or *H*. *ergaster* taxa [68,70,76–78]. In the present study, a broad *H*. *habilis* group was considered for all East African early *Homo* specimens dating between 1.7 and 1.4 Ma. No specimens from the Shungura and Koobi Fora formations, ascribed to the *Homo rudolfensis* clade [76,78–80], showed well-preserved buccal enamel surfaces, as was also the case for the occlusal surfaces [10,38]; therefore, the *H*. *rudolfensis* taxon was not considered. Due to the unresolved controversy concerning the *H*. *habilis* hypodigm from Olduvai [68], the Olduvai sample was considered as a single species [69]. The well-preserved *H*. *habilis* sample (N = 10) included seven specimens from Olduvai (OH 13, OH 16, OH 21, OH 27, OH 41, OH 62, and OH 69), one from Hadar (AL 666–1) and two from Omo (L 984 and 75s-69-14a) that have been considered as early *Homo* specimens [80,81]. The *H*. *ergaster* group included well-preserved specimens from Koobi Fora (ER 820, ER 992, ER 807, and ER 806), Olduvai (OH 23), and Nachukui (WT 15000). Three additional well-preserved remains from Koobi Fora (ER 1814, ER 3734, and ER 6128) have an uncertain *Homo* attribution [76,78,82,83] and were not included in the analysis. The paranthropine samples, dating from between 2.6 and 1.3 Ma [14,73], overlap both temporally and spatially with the early *Homo* group. The well-preserved specimens of this group included *P*. *aethiopicus* from West Turkana (WT 16005 and WT 17000) and Omo (L238-35, L338X-35, L62-17, and L860-2) and *P*. *boisei* from Koobi Fora (ER 1509, ER 1804, and ER 5431), Olduvai (OH 5 and OH 66), Nachukui (WT 17400 and WT 18600), Ileret (ER 729), Omo (L 7a-125), and Peninj (W64-160). The buccal microwear patterns of *A*. *anamensis* [65] (N = 5) and *A*. *afarensis* [66] (N = 26), as well as Cercopithecoidea [32,35] (N = 80) and Hominoidea primates [33] (N = 48), were used for comparative purposes (Table 1). Finally, a sample of *Theropithecus gelada* (N = 7, Natural History Museum, New York) from Ethiopia was also analysed, since its buccal microwear pattern was not yet available and this species has been proposed as a model for interpreting the diet of *Paranthropus* [84].

**Table 1:** The studied hominin fossil specimens by species. Hominin samples and comparative primate samples studied, indicating the number of specimens and teeth analyzed and the final well-preserved dental sample (exhibiting buccal enamel microwear features).

Samples studied	Specimens	Teeth	N
*Paranthropus aethiopicus*	44	46	7
*Paranthropus boisei*	56	158	10
*Homo habilis* (Early *Homo*)	49	153	10
*Homo ergaster*	18	89	6
*TOTAL hominines*	*167*	*446*	*33*
*Theropithecus gelada*			7
**Comparative samples** (31 hominins, 166 primates)			
*Australopithecus anamensis*			5
*Australopithecus afarensis*			26
*Mandrillus sphinx*			4
*Papio anubis*			27
*Chlorocebus pygerythrus*			15
*Cercopithecus mitis*			10
*Cercocebus torquatus*			3
*Colobus sp*.			21
*Gorilla gorilla gorilla*			31
*Gorilla beringei graueri*			7
*Pan troglodytes troglodytes*			10

Dietary habits greatly vary among the comparative primate samples studied. Geladas (*Theropithecus gelada*) are found in the high grassland of the deep gorges of the central Ethiopian plateau. They are the only primates that are primarily graminivores and grazers (grass blades make up to 90% of their diet). When both blades and seeds are available, geladas prefer the seeds, though they also eat flowers, rhizomes and roots when available [85]. Mandrills (*Mandrillus sphinx*) live in tropical rainforests and in gallery forests adjacent to savannas, as well as rocky forests, riparian forests, cultivated areas and flooded forests and streambeds. Forest-dwelling mandrills mostly feed (over 70% year-round) on mechanically protected plant foods such as hard-shell fruits or seeds from the ground [86,87], but will also eat leaves, lianas, bark, stems, and fibers; it also consumes mushrooms and soil [88]. The olive baboon (*Papio anubis*) is usually classified as savanna-dwelling, living in the wide plains of the grasslands, especially those near open woodland, but it also inhabits rainforests and deserts. The diet typically includes a large variety of plants, and invertebrates and small mammals, as well as birds. The olive baboons eat leaves, grass, roots, bark, flowers, fruit, lichens, tubers, seeds, and mushrooms, as well as corms and rhizomes that are especially important in times of drought. [89]. The main habitat of the vervet monkeys (*Chlorocebus pygerythrus*) is savanna woodlands. Its feeding habits consist of eating mostly fruits, vegetables, and small mammals, insects, and birds, making it an omnivore. The vervets needs to live around a source of water, especially during the dry season, and is able to adapt to many environments consuming a great variety of foods [90]. The blue monkey (*Cercopithecus mitis*) is found in evergreen forests, and lives largely in the forest canopy, coming to the ground infrequently. It is very dependent on humid, shady areas with plenty of water. They are primarily frugivores, with 50% of their diet consisting of fruit, with leaves or insects as their main source of protein, with the rest of the diet being made up of seeds, flowers, and fungi. They eat a variety of plants, but concentrate on a few species [91]. The collared mangabeys (*Cercocebus torquatus*) are found in coastal, swamp, mangrove, and valley forests. It has a diet based of fruits (60%) and seeds (20%), but also eats leaves, foliage, flowers, invertebrates, mushrooms, dung, and gum [92]. They have thick enamel to process hard-object foods as a fallback feeding strategy, such as bark or seeds, when preferred foods (fruits) are unavailable [93]. The Colobus monkeys (*Colobus sp*.) are arboreal [94], traditionally classified as a genuine leaf-eaters [95], but are considered to have a heterogeneous diet, including fruit, flowers, and twigs [96]. Their habitats include primary and secondary forests, riverine forests, and wooded grasslands. Their ruminant-like digestive systems [97] have enabled these leaf-eaters to occupy niches that are inaccessible to other primates [98]. Finally, within the hominoidea primates, the western lowland gorillas (*Gorilla gorilla gorilla*) live in primary and secondary rain forests and lowland swamps in central Africa. They eat a combination of fruits and foliage depending on the time of year. When ripe fruit is available, they tend to eat more fruit as opposed to foliage. When ripe fruit is in scarce supply, they eat leaves, herbs, and bark. Gorillas choose fruit that is high in sugar for energy, as well as fiber, and in the dry season they still continue to eat other kinds of fruits, and they may also eat insects from time to time [99]. The Grauer’s gorilla (*Gorilla beringei graueri*) is endemic to the mountainous and lowland forests of eastern Democratic Republic of Congo. They prefer fruits, but when scarce they increase the consumption of leaves, pith, and barks [100]. The common chimpanzee (*Pan troglodytes*) lives in a variety of habitats, including dry savanna, evergreen rainforest, swamp forest, and dry woodland-savanna mosaic. It is an omnivorous that prefers fruit above all other food items and even seeks out and eats them when they are not abundant. It also eats leaves and leaf buds, as well as seeds, blossoms, stems, pith, bark and resin. Insects and meat make up a small proportion of their diet [100,101].

### Sample selection and processing

**Fig 1:**
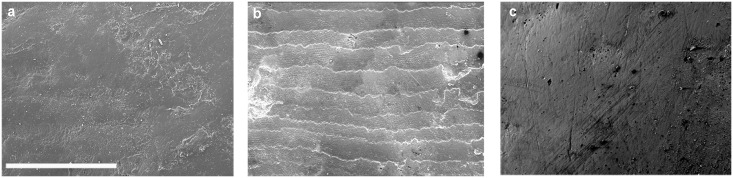
SEM images of *post-mortem* damaged teeth that were not included in the buccal microwear analyses. (a) LP^4^ OH-65 with patina layers covering the microwear features. (b) LM_1_ KNM-ER-1171 with perykimata—growth lines—and enamel prisms caused by chemical erosion. (c) RP^4^ OH-5 with *post-mortem* physical abrasion caused by rolling over sediments. Scale line is 200 μm.

**Fig 2:**
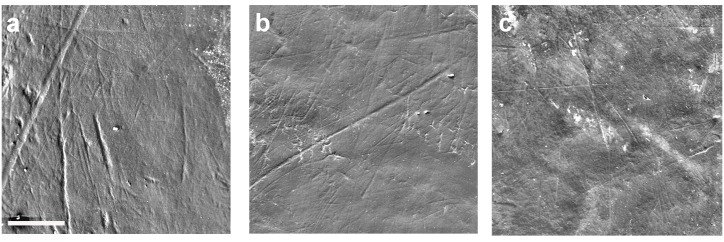
Well-preserved buccal microwear surfaces in which buccal striations could be measured. (a) LP_4_ OH-69 *Homo habilis*. (b) RM_1_ KNM-WT-15000 *Homo ergaster*. (c) LM_1_ Peninj *Paranthropus boisei*. Scale line is 200 μm.

Taphonomy may severely limit available samples [18]. The fossil teeth analyzed showed considerable *post-mortem* damage (Fig 1) that included chipping, surface erosion, etching, and weathering patterns, similar to that previously described for Olduvai and Hadar specimens [102]. The damage was initially attributed to the consumption of acidic foods [103,104] but was later thought more likely to be related to *post-mortem* wear affecting the entire tooth dental crown, including the inter-proximal wear facets [54,58]. *Post-mortem* damage was also observed on the occlusal surfaces of the same specimens [40]. The East African fossil specimens unearthed at ancient paleo-lakes and fluvial areas [4,105–107] were probably damaged by prolonged surface exposure or by water transport [38]. Such circumstances are likely to be responsible for most of the *post-mortem* abrasions observed, and such abrasions were also present in some unworn, not-fully functional teeth [102]. The well-preserved (Fig 2) samples consisted of 66 teeth (14.8% of the 446 teeth studied) belonging to 36 hominin specimens. This low preservation rate is similar to those described for buccal enamel surfaces in *A*. *anamensis* and *A*. *afarensis* specimens from Hadar [65,66], as well as to those observed for occlusal surfaces in the same specimens [19,38].

The methods for sample selection followed standard procedures in buccal microwear research [41–43,62]. A single tooth, showing a well-preserved buccal enamel patch, was chosen to represent each individual. The lower M_1_, either left or right, was preferentially selected when available because it is the first tooth to erupt. Otherwise, P_4_, M_2_, P_3_, or M_3_ were selected (in that order) in preference to the upper dentition. Consequently, the final studied sample included 33 specimens from the 66 well-preserved teeth belonging to the four hominine species studied: 7 *Paranthropus aethiopicus*, 10 *Paranthropus boisei*, 10 *Homo habilis*, and 6 *Homo ergaster*, as well as 3 undetermined *Homo sp*. specimens from Koobi Fora (Table 2).

**Table 2:** Dental sample showing well-preserved buccal microwear patterns. Specimens numbers and paleontological information (hominin species, stratigraphic site complex and unit) are provided for all the teeth showing well-preserved buccal microwear patterns.

Species	Complex	Unit	Specimen	Tooth
*P. aethiopicus*	Omo	C	L 62–17	RM_2_
		F	L 157–35	M_2_
		F	L 238–35	M^2^
		E	L 338x-35	P^3^
		F	L 860–2	RM_2_
	West Turkana	Middle D	KNM-WT 16005	LM_1_
		Lokalalei	KNM-WT 17000	LM^2^
*P. boisei*	Nachukui	Kaitio	KNM-WT 17400	RP^4^
		Natoo Mb	KNM-WT 18600	LP^3^
	Koobi Fora	KBS	KNM-ER 1509	LM_2_
		Upper KBS	KNM-ER 1804	RM^1^
		Tulu Bor Mb	KNM-ER 5431	RM_1_
	Ileret	Okote	KNM-ER 729	RP_4_
	Omo	Shungura G	L 7a-125	RP^4^
	Olduvai	Bed I	OH 5	RM^1^
		Upper Bed I	OH 66	LP_4_
	Peninj	Humbu F	NMT-W64-160	LM_1_
*Homo habilis*	Hadar	BKT-3	AL 666–1	LM^2^
	Olduvai	Bed II	OH 13	LM^1^
		Lemuta Mb.	OH 16	RM_2_
		Surface	OH 21	LM^1^
		Bed I	OH 27	RM_1_
		Bed II	OH 41	LM^1^
		Bed II	OH 62	RM^2^
		Bed II	OH 69	LP_4_
	Omo	Shungura G	L 984	RM^1^
		Shungura G	75s-69-14a	LP_3_
*Homo ergaster*	Koobi Fora	Okote Mb.	KNM-ER 806	LM_1_
		Okote Mb.	KNM-ER 820	LM_1_
		Okote Mb.	KNM-ER 992	RM_1_
		KBS	KNM-ER 807	RM^3^
	Nachukui	Natoo Mb.	KNM-WT 15000	RM_1_
	Olduvai	Bed IV	OH 23	LP_4_
*Homo sp.*	Koobi Fora	Upper KBS	KNM-ER 1814	LM_1_
		Upper Burgi	KNM-ER 3734	LM_2_
		KBS	KNM-ER 6128	RP^4^

### Dental casts and scanning electronic microscopy observations

Dental crown molds were made with President MicroSystem Regular Body (Coltène^™^) polyvinyl siloxane following standard procedures [108–110]. Positive casts were made with epoxy resin or polyurethane (Epotek 301 Epoxy Technologies, Inc. Billerica, MA) following the manufacturer's indications. All casts were mounted on aluminum stubs and sputter-coated with a 40 Å gold layer. Prior to scanning electronic microscopy (SEM) examinations, all replicas were observed under a binocular light microscope at 10–30× magnification. The replicas that exhibited clear *post-mortem* damage [102], such as multiple parallel scratches, enamel chipping or enamel prisms exposure, both at low magnification or under SEM observation [18], were discarded to prevent non-dietary related factors from affecting the buccal microwear pattern analysis.

Well-preserved buccal enamel surfaces were digitized under SEM at the *Centres Científicos i Tecnològics* (CCiT) of the Universitat de Barcelona, at 100× magnification, 18–25 mm working distance (WD), and 15 kV acceleration voltage [111,112]. Slight variations in WD did not affect the measurements of the microwear features because all analyzed images were cropped to exactly cover 0.56 mm^2^ (748.33 × 748.33 μm) of enamel surface, the standard dimensions used in SEM buccal microwear studies [41–43,62], and all image measurements were scaled prior to analysis. During scanning, the buccal enamel surface of each tooth crown was placed perpendicular to the electron beam, with the occlusal crown rim facing upwards in all SEM images. The digital images were taken in the middle third of the crown, avoiding both the occlusal and cervical thirds [42]. A high-pass (50-pixel) filter and the automatic grey levels adjustment command in Photoshop 7.0 (Adobe^™^) were applied to all cropped digital grey-scale images to reduce shadows and enhance image contrast.

### Analysis of buccal microwear patterns

In the selected micrographs, the length (in μm) and orientation angle (with respect to the horizontal occlusal plane) of all observed scratches within the studied enamel patch (including those cropped by the observation area) were measured using a semi-automatic procedure with SigmaScan Pro 5.0 (SPSS^™^) software. All scratches measuring less than 10 μm in length (approximately 4 times the average width) were not considered [42]. Following standard procedures in buccal microwear research [41,42,43,62], measures of the density of scratches (N) and their average length (X) were obtained for all the observed striations by four 45°-degree orientation categories −vertical (V), horizontal (H), mesio-occlusal to disto-cervical (MD), and disto-occlusal to mesio-cervical (DM)−, as well as for the total number of striations observed (T) (see Methods section). Consequently, a total of 10 variables were derived for each tooth studied (NV, XV, NH, XH, NMD, XMD, NDM, XDM, NT, XT) (S1 Table), so that the interpopulation differences could be referred to specific striation densities and lengths by orientations categories. While all the studied variables exhibited normal distributions (Kolmogorov-Smirnov tests, P > 0.05) for all the hominin groups considered (Early *Homo*, *H*. *ergaster*, *P*. *aethiopicus*, *P*. *boisei*), rank-transformed variables were used for the inter-group comparison analyses because sample sizes were small and differed greatly among groups, as well as bevause.

Inter-observer error is a major concern in microwear research both for the occlusal [62] and buccal [63] enamel surfaces. Therefore, a single observer (LMM) measured all the micrographs of the fossil hominins studied, as well as of the *Theropithecus* specimens used for comparison. However, the primate comparative samples [33,113] and the *A*. *anamensis* and *A*. *afarensis* specimens [65,66] were measured by different researchers (JG and FE, respectively). Nonetheless, the inter-observer error analyses among all the researchers involved in this study have not shown significant differences in the buccal microwear patterns measured [60].

Comparisons of buccal microwear patterns among the hominin groups studied were made with a multiple analyses of variance (MANOVA) and *post-hoc* pairwise comparisons with Bonferroni correction of *P*-values. The variability in the dispersion of buccal microwear patterns among these hominin groups and among the hominines and the comparative samples are illustrated with Linear Discriminant Analysis (LDA) plots of the first two discriminant functions obtained (DF1 *vs*. DF2), using the species as the independent variable to determine if species with similar microwear patterns show similar diets or share similar environmental conditions. A Cluster Analysis (CA) of the group centroids was derived from the diagonal matric of Fisher's distances among the groups obtained in the CVA. All the statistical analyses and group comparisons were made using PAST v. 3.10 statistical package [114] and XLSTAT v. 2015 (Addinsoft).

## Results

**Fig 3:**
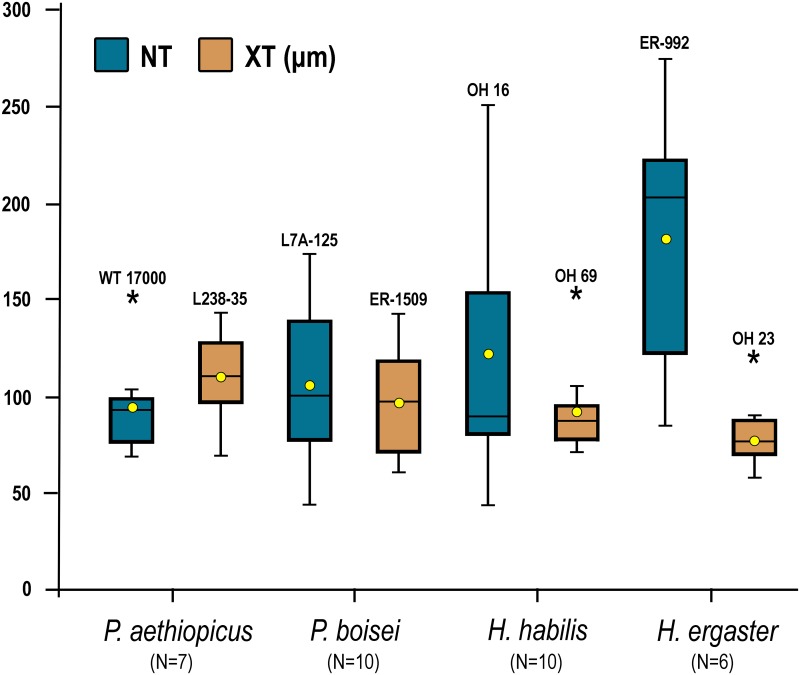
Box plots of microwear total striation density and average striation length by species. The whiskers show the minimum and maximum values (excluding outliers). The box includes the 25–75 percentiles. Both the median values (lines within the boxes) and means (yellow dots) are shown for the total striation density (NT) and length (XT) by species (sample sizes are indicated in brackets). For the outliers the specimen reference numbers are shown.

Average total striation density (NT) is smaller in *Paranthropus aethiopicus* (N = 7, NT = 94.9) and *P*. *boisei* (N = 10, NT = 105.9) than in *H*. *habilis* (N = 10, NT = 122.3) and *H*. *ergaster* (N = 6, NT = 181.8). Both *Paranthropus* taxa show larger average striation lengths (XT) than the *Homo* taxa (Table 3, Fig 3), although there were no significant differences in striation density or length (rank-transformed data) between the four groups (MANOVA Wilks' lambda = 0.401; F = 0.994; P = 0.486). The four hominines studied showed fewer average striation densities than *A*. *anamensis*, and only *H*. *ergaster* showed more scratches than *A*. *afarensis*.

**Table 3:** Average values of the 15 microwear variables analyzed for each taxonomic group considered. N: Sample Size; the Total Number of Striations (N), Average Length of All Striations (X), and Standard Deviations of the Length (S) are Indicated by Orientation: Horizontal (H), Vertical (V), Mesio-distal (MD), Disto-mesial (DM), and All Striations (T).

Species	*N*	NH	XH	NV	XV	NMD	XMD	NDM	XDM	NT	XT
*A*. *anamensis*	5	36.8	90.4	68.0	110.5	77.4	86.5	26.6	87.3	208.8	97.4
*A*. *afarensis*	26	41.8	111.9	28.3	124.2	39.4	109.3	47.4	93.9	150.7	111.6
*P*. *aethiopicus*	7	25.7	105.8	17.6	135.1	17.7	80.7	33.9	101.2	94.9	110.5
*P*. *boisei*	10	24.0	96.4	21.8	139.4	32.6	76.7	27.5	99.1	105.9	97.1
*H*. *habilis*	10	24.6	106.6	21.6	108.2	23.2	81.5	52.9	76.4	122.3	92.6
*H*. *ergaster*	6	51.7	81.4	28.8	111.2	66.5	72.3	34.8	66.4	181.8	82.0
*P*. *troglodytes*	10	52.8	92.3	40.4	126.9	38.8	79.0	39.9	94.3	171.9	98.3
*G*. *gorilla gorilla*	31	40.1	83.1	53.3	128.9	44.3	83.6	47.0	91.0	184.7	100.8
*G*. *beringei graueri*	7	34.3	87.3	44.3	162.6	33.7	82.1	45.3	85.7	157.6	109.6
*Cercocebus torquatus*	3	52.7	73.4	45.7	109.0	66.7	68.1	84.3	81.6	249.3	82.1
*Cercopithecus mitis*	10	34.8	83.9	108.8	99.8	60.6	74.4	39.8	76.0	244.0	87.4
*Chlorocebus pygerythrus*	15	34.7	73.2	81.0	112.1	56.4	75.2	38.5	80.9	210.6	90.3
*Colobus sp*.	21	42.5	79.4	35.6	112.2	37.0	74.9	30.6	85.3	145.7	89.4
*P*. *anubis*	27	21.4	90.8	85.8	118.3	49.8	88.8	20.4	86.2	177.5	103.7
*Mandrillus sphinx*	4	46.0	77.7	60.3	116.8	54.5	86.2	53.0	78.4	213.8	92.4
*Theropithecus gelada*	7	21.4	61.9	79.0	116.3	44.1	79.6	32.1	75.6	176.7	91.7

**Fig 4:**
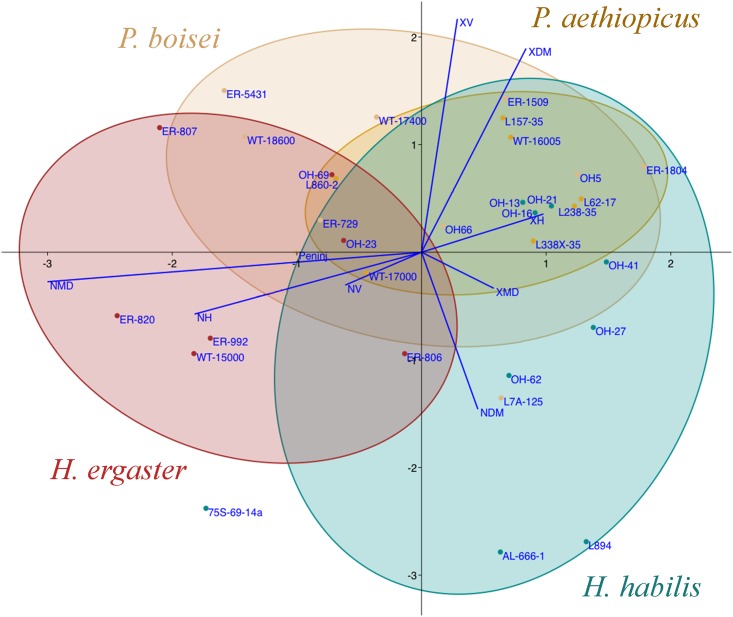
Plot of DF1 on DF2 derived from the Linear Discriminant Analysis of the buccal microwear variables of the hominin groups studied. Plot of the first two discriminant functions (DF1 x axis, DF2 y axis), derived from the microwear variables (ranked data) for the hominines samples studied (*Paranthropus aethiopicus* brown, *Paranthropus boisei* beige, *Homo habilis* cyan, *Homo ergaster* red), that explain 93,8% of the total variance (55.9% and 37.9%, respectively). The ellipses show one standard deviation of the sample means (68% confidence interval of the sample). The blue lines represent the loadings of the microwear variables on the discriminant functions. The analysis was made with PAS v. 3.

The linear discriminant analysis (LDA) of the four hominin groups studied (*P*. *aethiopicus*, *P*. *boisei*, *H*. *habilis*, *H*. *ergaster*) was able to correctly classify 51.5% of all cases, although that ability diminishes to only 24.2% after jack-knife cross-validation. The first two discriminant functions (Fig 4) explain 93.8% of the total variance (55.9% DF1 and 37.9 DF2). DF1 shows negative loadings for NMD (−35.8), NH (−21.7) and NV (−7.3) and positive loadings for XH (11.7), XDM (10.0), XMD (6.9), and XV (3.4), as well as for NDM (5.4), which behaves differently in this respect than NMD. DF2 shows negative loadings with all the density variables (mainly with NDM, −17.4), as well as with XMD (−4.0), and shows positive loadings with all the length variables (mainly with XV, 25.8, and XDM, 22.6). Despite the four groups greatly overlap, *Homo ergaster* specimens show negative values for DF1 that reflect their higher striation densities compared to the other taxa. A *post-hoc* classification of the ungrouped *Homo* sp. specimens (ER-1814, ER-3734 and ER-6128) within the LDA showed that their buccal microwear pattern is most similar to that of *H*. *habilis*, with a 100% *post hoc* classification probability in all three cases.

**Fig 5:**
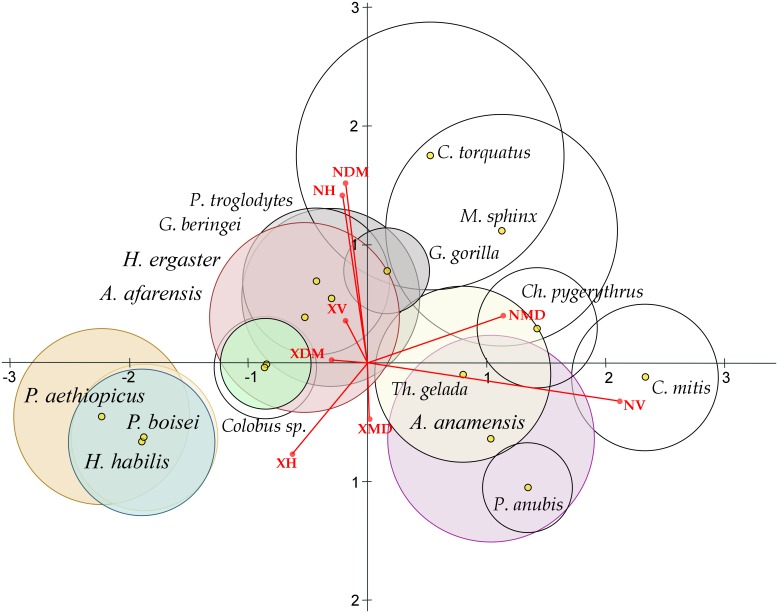
Plot of DF1 on DF2 derived from the Linear Discriminant Analysis Analysis of the hominines studied along with all the comparative samples. Plot of the first two discriminant functions (DF1 x axis, DF2 y axis), derived from the microwear variables (ranked data) for all the specimens studied and the comparative collections, that explain 73,66% of the total variance (57,25% and 16,41%, respectively). The circles represent the 95% confidence intervals of the group centroids assuming equality of covariance matrices (the size of the circle depend on the sample sizes). The red lines indicate the correlations between the variables considered and the two functions shown. The analysis was made with XLSTAT v. 2015.

The second discriminant analysis, including all the comparative samples, expands the microwear pattern diversity observed in the LDA of hominines alone. The multiple MANOVA test within the LDA clearly shows significant among-group differences (Wilks' lambda = 0.133, F = 3.3454, P<0.0001; S2 Table), and the *post hoc* pairwise comparisons using Fisher's distance (d_F_) between groups show significant differences between *H*. *ergaster* and the two *Paranthropus* taxa (*P*. *aethiopicus* d_F_ = 2.755, P = 0.007; *P*. *boisei* d_F_ = 2.272, P = 0.025), but not between *H*. *habilis* and the paranthropines (*P*. *aethiopicus* d_F_ = 1.067, P = 0.388; *P*. *boisei* (d_F_ = 1.794, P = 0.081). Significant differences were also found between *H*. *habilis* and *H*. *ergaster* (d_F_ = 2.775, P = 0.006), though not between *P*. *aethiopicus* and *P*. *boisei* (d_F_ = 0.431, P = 0.902). The four hominines could be significantly discriminated from *A*. *afarensis*, while *H*. *ergaster* was the only group that did not significantly differed from *A*. *anamensis* (d_F_ = 1.533, P = 0.149). The univariate among-groups comparisons (ANOVAs) were significant (P<0.03) for all the variables studied except XDM (F = 1.016, P = 0.441; S3 Table). The scatterplot of DF1 vs. DF2 (Fig 5) explains 73,66% of the total variance (57,25% and 16,41%, respectively; S4 Table), and 46,73% of the 199 specimens were correctly classified, a value that diminished to 31.16% after jack-knife cross-validation. DF1 was highly correlated with NV (r = 0.922) and NMD (r = 0.494), whereas DF2 was correlated with NDM (r = 0.706), NH (r = 0.663), XH (r = −0.359), and XMD (r = −0.214) (S5 Table). Only 46.73% of all cases were correctly classified (S6 Table) and this figure decreased to 31.16% after jack-knife cross-validation (S7 Table). The hominin taxon with the highest posterior classification probability before validation is *H*. *habilis* (60,0%), followed by *P*. *boisei* (50,0%), *H*. *ergaster* (16.7%), and *P*. *aethiopicus* (14.3%). In fact, the two paranthropines greatly overlap in the LDA (Fig 5), and 64.3% are correctly classified into a robust taxon. The *Homo habilis* specimens overlap with the paranthropines for both DF1 and DF2, whereas *H*. *ergaster* shows a distinct distribution along both DF1 and DF2, overlapping with the hominoidea primates and with *Colobus*. As has already been shown [43], *A*. *anamensis* specimens more closely resemble the cercopithecoidea primates, especially *Papio anubis* and *Theropithecus gelada*, in having high striation densities, mainly the vertical ones (NV), with the exception of *Colobus* that has a smaller striation density that overlaps for DF1 with the hominoids, likely due to its mainly folivorous diet compared to the other cercopithecoidea taxa studied that consume greater amounts of hard foods, especially, seeds [32,33]. The largest numbers of vertical striations (NV) are observed in *Cercopithecus* (108.8), followed by *Papio* (85.8), *Chlorocebus* (81.0), *Theropithecus* (79.0), *Mandrillus* (60.3), and *Cercocebus* (45.7), with *A*. *anamensis* showing a NV value (68.0) well within the range of these cercopithecoidea samples. Despite the diet of *C*. *mitis* is mainly composed of fruits, this species showed the highest density of vertical striations (NV) and one of the highest total striation densities (NT = 244.0) observed, along with *C*. *torquatus* (NT = 249.3), which suggests that they might have also relied on harder items, perhaps as fallback foods. The hominoidea primates considered show significantly lesser NV values (53.3 *G*. *gorilla gorilla*, 44.3 *G*. *beringei graueri*, and 40.4 *P*. *troglodytes*) that overlap with that of *Cercocebus* (45.7). *Colobus* (35.6) shows a NV values similar to that of *A*. *afarensis* (28.3), while the other hominins studied show significantly smaller values (28.8 *H*. *ergaster*, 21.8 *P*. *boisei*, 21.6 *H*. *habilis*, and 17.6 *P*. *aethiopicus*). Within the cercopithecoidea, the highest values of NDM are observed in *Cercocebus* (84.3) and *Mandrillus* (53.0), and the lowest is seen in *Papio* (20.4). However, the cercopithecoidea and hominoidea greatly overlap for DF2 (Fig 5). *Theropithecus* has been proposed as a model for interpreting the diet of *Paranthropus* [84]. However, its overall average striation density (NT = 176.7) is much higher than those of both *P*. *aethiopicus* (94.9) and *P*. *boisei* (105.9) −in fact these are the smallest average striation densities observed in all the samples studied−, and both hominins significantly differ from the primate taxon (d_F_ = 4.434, P<0.0001 for *P*. *aethiopicus*; d_F_ = 4.249, P = 0.000 for *P*. *boisei*), despite the fact that their habitats might not have differed greatly from those occupied by contemporaneous *Theropithecus*.

**Fig 6:**
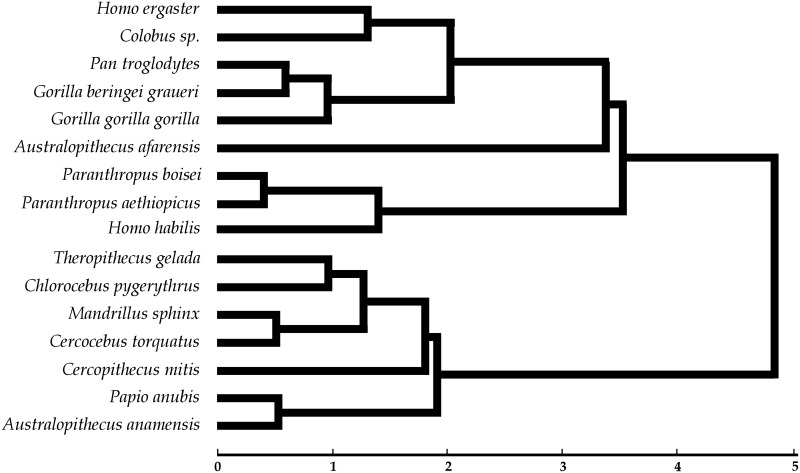
Phenetic dendrogram of similarities among group centroids of all the samples considered. The dissimilarities among groups were measured using Fisher's distance derived from the Linear Discriminant Analysis of all the microwear variables (ranked data) for all groups considered. The diagonal dissimilarity matrix was used to derive a hierarchical cluster analysis using an unweighted average agglomeration method in XLSTAT v. 2015.

Finally, a hierarchical cluster analysis (CA) was obtained (Fig 6) for all the taxonomic groups considered using Fisher's measure of distance (dissimilarities) derived from the CVA (S8 Table). The dendrogram shows that *H*. *ergaster* clusters with *Colobus*, and the two taxa group with the chimpanzees and gorillas, whereas *H*. *habilis* clusters with both *Paranthropus* taxa. A significant separation (S9 Table) can be observed between *A*. *anamensis*, which groups with *Papio*, and then with the rest of cercopithecoidea primates studied (except *Colobus*), and *A*. *afarensis* that clusters within the hominoidea taxa that also includes *Colobus*.

## Discussion

Phytoliths require some 6,000 mega-Pascal (MPa; 6,000 mega-Pascal = 6,000 Newton/mm^2^) of force per unit area to deform [115]. It has been suggested that enamel can be scratched at about half that force (3,700 MPa) [116]. Based on this estimate, it has been argued that the forces applied to food on the buccal side of a tooth may not be sufficient to scratch enamel [18], indicating that it is "merely a matter of believe" that as food matter is masticated the abrasive particles scratch the buccal enamel surfaces (page 290 in [18]). However, more recent estimates suggests that the critical loads required to fracture enamel in humans exceed 500 Newtons (N), whereas the loads required to produce individual microwear traces are in the order of milli-Newtons (mN), less than 1 N per unit area (<1 MPa) [117] −much less that previously suggested 3,700 MPa. Whatever the case, our experimental analyses on modern human volunteers have clearly shown that striations on buccal enamel surfaces are in fact formed as a result of food chewing [49], despite the buccal side of the tooth is not involved in tooth-to-tooth contact during food processing. Experimental analyses have shown not only that buccal enamel scratching is possible, but also that despite scratch formation rates on buccal surfaces are faster than expected, buccal microwear patterns can be used as long-term proxy for inferring dietary habits [50,51]. Furthermore, buccal microwear patterns have been shown to significantly discriminate among dietary habits of living hunter-gatherer pygmy populations from western central Africa with varying degrees of hunting and gathering practices, as well as from agricultural populations [63]. Despite these evidences, buccal microwear incidence has also been suggested to be "most likely related to differences in the frequency of terrestrial feeding events" in relation to the "opportunity to ingest exogenous grit" [18]. However, the volunteers in our experimental buccal microwear analyses [49,63] are not likely to be consumers of grit and dust. In addition, complex behaviors, such as the possibility that hominins could have cleaned dirty food before consuming it, with or without stone tools, should not be disregarded. An occlusal-limited view of attritional processes disregards the fact that the energy of a particle depends not only on the forces applied on occlusal surfaces, either involving no particle movement (causing pits) or slight and angular displacement (causing scratches). It also depends on the mechanical energy attributable to particles, especially their speed, while moving between the cheek and the buccal enamel surfaces of teeth during food chewing. As has been acknowledged, the precise causes of microwear formation are difficult to "pin down" and involve mechanics that are more complex than imagined [118]. More specific research on enamel etching, analyzing both forces and particle speed, are yet required.

In addition, attempts to associate buccal microwear patterns of fossil hominins to post-depositional, taphonomic processes should also be valid for occlusal enamel surfaces. There is no reason to disregard well-preserved, *ante-mortem* microwear signals, either on buccal or occlusal dental surfaces, since those microwear features have been observed on live modern humans, as well as primates. It is germane to note that taphonomic processes affect both buccal and occlusal enamel surfaces and that *post-mortem* damage can be properly dealt with in both cases [18,102].

Occlusal enamel microwear SEM or texture data are available for most African hominin species [18,19,38,67,119,120], whereas buccal microwear data for the same specimens is yet scarce [65,66]. Consistent dietary interpretations of both occlusal and buccal enamel surfaces analyses have been obtained for *Australopithecus anamensis* specimens [65,120], whereas contrasting results have been shown for *Australopithecus afarensis* fossil specimens [66,119]. The occlusal microwear pattern of *A*. *afarensis* have suggested that *Gorilla* and/or *Theropithecus* constitute the best modern analogues for dietary preference of this species, and that there is no occlusal microwear evidence of the mastication of hard, brittle items [118]. The buccal microwear pattern of *A*. *afarensis* has shown resemblances in striation densities, although not in striation lengths, to those of *Gorilla* and *Pan*, which are clearly distinct from those of cercopitecoid primates from more open environments [66]. In the present study, *A*. *afarensis* specimens show a distinct buccal microwear pattern that significantly differs from those of most of the cercopitecoids species studied (except *Colobus*), including *Theropithecus* that was not available in our previous analyses. The buccal microwear patterns of *A*. *afarensis* and *Theropithecus* significantly differ (d_F_ = 4.712, P<0.0001), which suggests that the diet of *A*. *afarensis* was not heavily dependent on grasses or seeds. It would have rather consumed greater amounts of less abrasive foods, such as fruits and foliage, since its buccal microwear pattern more closely resemble that of the hominoids that those observed in the cercopitecoids. By contrast, *Australopithecus anamensis*, characterized by a high density of vertical scratches, greatly overlaps with *Theropithecus* in the CVA and the two taxa do not significantly differ (d_F_ = 1.192, P = 0.307). Our analysis supports that the diet of *A*. *anamensis* would have significantly relied on seeds and grasses from open woodlands, in addition to fruits from more closed environments, as we have previously suggested [65].

Fossil specimens of *P*. *aethiopicus* have shown high carbon isotope ratios (δC^13^) suggestive of a significant consumption of C4 or/and CAM plant foods (≥ 50%)—clearly higher (with specimen WT-17000 being an outlier) than those shown by *A*. *anamensis*, which would have preferred C3-rich diets with low percentages of C4 plants [121–123]. Nevertheless, *P*. *aethiopicus* shows the lowest overall density of buccal scratches, with WT-17000 showing the highest striation density compared to the other six specimens of the same taxon (see raw data in S1 Table). *Paranthropus boisei* specimens have also shown high C4 isotopic signals, ranging from 75% to 80% [20,121]. However, their buccal microwear patterns do not support an abrasive diet based on hard, tough, or fibrous C4 plant foods, a result concordant with that obtained on occlusal surfaces [19]. In contrast, the significance of CAM plants such as the succulent xerophytes, which would be less abrasive but result in greater C4 signals, is difficult to disentangle. The significant C4 signal of *P*. *boisei* could also be due to secondary C4-based diet from animal foods [124] or to consumption of aquatic resources [125], which is supported by the δO^18^ values [17]. The buccal microwear patterns of *P*. *aethiopicus* and *P*. *boisei* do not support the hypothesis of consumption of highly abrasive or tough foodstuffs, in contrast to the assumption that their distinct cranio-dental adaptations are indicative of highly abrasive food intake, mostly USOs [126]. The robust facial and dental anatomy of *Paranthropus* might reflect the occlusal biomechanics of food processing (such as peak loads, repeated loadings, and tooth-food-tooth contact while chewing) as the cause of enamel fracture rather than the chewing of abrasive foods. The preparation and ingestion of large, fracture-inducing food objects such as nuts and seeds might result in distinct, high-density occlusal microwear signals [127,128]. In contrast, buccal microwear patterns might depend more on the particle movements during the chewing cycle rather than on food cutting, cracking, or grinding with dental occlusal surfaces. Low cheek-to-tooth loadings and the kinetics of abrasive particles in the mouth, along with the amount of chewed abrasives, sufficiently explain scratch formation on buccal enamel surfaces [49]. Thus, buccal microwear is more indicative of food properties than occlusal microwear, which is also dependent on the mechanics of the chewing cycle. Biomechanically challenging USOs (underground storage organs) such as corms, seeds, or bulbs could lead to isotopic signals that would match the low microwear feature densities observed in *Paranthropus boisei* [129]. Moreover, C4 resources capable of causing high densities of microwear features remain to be found in East Africa [130].

A durophage-ecotone trophic model [124] for the robust australopithecines would be consistent with the buccal microwear patterns, morphological adaptations, and isotopic indicators. If *P*. *boisei* did not consume highly abrasive diets, its robust dental morphology and thick enamel could have developed as adaptations to consumption of hard-shelled invertebrate prey such as crabs, similar to those present in animals such as marsh mongoose (*Atilax*) or Cape clawless otter (*Aonyx*) that live in riverine or lake environments [37,131]. A durophage-ecotone model for the robust australopithecines was suggested to provide a clear mechanism, based on habitat and trophic preferences, to explain the long-term coexistence of *Paranthropus* and early *Homo* [124]. A durophage dietary hypothesis for the paranthropines would also be consistent with the observed microwear results, the morphological adaptations [111], and the isotopic indicators [132]). However, it has been questioned whether *P*. *boisei’s* dental traits would have evolved as an adaptation to a diet specialized on freshwater crabs. Grine *et al*. [18] argued that otters would not serve as a model for hard-object feeding in *P*. *robustus* due to its thin enamel. Moreover, there are concerns about the viability of a diet specialized in freshwater resources. Ancient hominin species lived near freshwater springs, rivers, lakes or estuaries [133–137], which has led several authors to speculate that access to freshwater from endorheic lakes of the East African Rift System (EARS) would have been vital for hominin survival and expansion during the Pliocene [134,138–140]. However, many of these lakes would have been saline during the Pliocene [140,141]. It may seem unlikely that *P*. *boisei* would have been a crab-specialist in a highly dispersed resource landscape if the productivity of the freshwater lakes would not have been sufficient to maintain the large populations of crabs needed to feed a highly specialized crab-consuming hominin [133]. However, consumption of fresh water crabs might have been a sporadic, or even a fallback resource for the paranthropines.

Early *Homo* specimens have shown isotopic signals mainly indicative of C3 plant consumption (45−65%) with an increase in C4 resource consumption of more than 20% over time [122], which is consistent with the observed increase in buccal microwear striation densities from *H*. *habilis* to *H*. *ergaster*. On one hand, early *Homo* specimens show low scratch densities suggestive of chewing soft foods, consistent with meat consumption as a major dietary source and increased brain volume [28,142]. On the other hand, *H*. *ergaster* specimens show higher and more variable striation densities, suggestive of consumption of a wider range of hard and/or tough items than early *Homo* and consistent with an ecological diversification of the *Homo* clade [143]. Although more research is needed to clarify the question of the synchrony of two, or even three, *Homo* species, the distinct buccal microwear signals observed in early *Homo* and *H*. *ergaster* specimens suggest a clear temporal trend in food exploitation strategies that might be related to increasing dependence on mechanically demanding USOs [129]. An efficient exploitation of a wide range of high-quality fallback foods might have required complex behavioral adaptations, including the use of tools for food processing [39]. The evolved Acheulian lithic industry linked to *H*. *ergaster* might have provided the opportunity and flexibility required to differentially exploit a variety of fallback resources, although it might have not significantly reduced the amount of abrasive particles in foodstuffs. Climatic fluctuations in East Africa approximately 1.8 Ma and the aridity peak coincident with the emergence of *H*. *ergaster* [3] might explain its highly abrasive buccal microwear signal, which is indicative of a more abrasive diet in this taxon than in early *Homo*—a finding that is in line with an omnivorous diet including plant foods with silica particles [40] that are abundant in East African open and arid environments [144]. This does not rule out the hypothesis of meat as an important food source, but dependence on plant foods would have been at least of similar importance as that described for modern hunter-gatherer populations from arid environments [129,145]. Despite the occlusal crown relief suggesting that *H*. *habilis* would have heavily relied on fallback resources [146], the buccal microwear patterns suggest that early *Homo* might have ingested fewer abrasives than *H*. *ergaster*, which may have depended on harder and tougher fallback foods. Differences in the exploitation of ecological niches by the two species may explain the buccal microwear differences observed: wooded, gallery forest with softer foods rich in sugars by *H*. *habilis* and open savannas with abrasive food items by *H*. *ergaster*, irrespective of the amount of meat they consumed.

Finally, differences in temporal scales of dietary proxies [147] might be relevant to explain the lack of concordance of dietary interpretations, especially between isotope data, indicative of dietary practices during the time of dental crown development, and occlusal microwear and texture data, largely affected by the "last-supper" effect. Carbon and oxygen isotopes are incorporated into the teeth during the formation and mineralization of the enamel (from a few months to two years). The dietary signal is therefore averaged over that time period and for the time that enamel becomes fully mineralized. Thus, the enamel might not reflect the animal's diet right before death. In contrast, the rate of formation of microwear features on occlusal surfaces is so fast that microwear patterns and texture measures might be reflecting the diet consumed only a few days before death, which has been referred to as the "Last Supper Effect". Despite microwear traces of diet might be somewhat ephemeral on occlusal surfaces, one can decipher the actual dietary habits of an individual at a given point [18]. However, buccal microwear patterns might be informative of a longer span of dietary-related activities. The rates of newly formed features per week may increase from about 3% in normal *ad-libitum* diets to 9% when dried meat or fish are consumed, or to 22% when stone-ground flower is consumed. In the short span of about 5 weeks of this experimental design, there was no loss of microwear features. However, in the long-term experiments, no significant increase through time () was observed in the striation densities, and the average net long-term turn over rate on buccal enamel surfaces was −0.009 scratches/week. This suggests a stasis in microwear patterns depending on dietary habits or ecological factors [49]. Although buccal microwear patterns might average a longer period of dietary habits than occlusal microwear patterns, they are more likely to show concordant results than if compared to carbon isotopic stages if the diets of infant and adults hominins differed.

## Conclusions

Patterns of buccal dental microwear striation densities and lengths are consistent with enamel microwear complexity and anisotropy on occlusal dental surfaces previously described for East African Lower Pleistocene *Paranthropus* specimens. Our results do not support the dietary interpretations based on 13C stable isotopic ratios that suggest a significant consumption of C4 plant foods in open environments. Quite the contrary, the buccal microwear patterns suggest that the dietary habits of both *P*. *aethiopicus* and *P*. *boisei*, unlike early *Homo* and *H*. *ergaster*, did not involve chewing significant amounts of abrasive foods. Alternatively, consumption of non-abrasive, though brittle, C4-rich resources would be consistent with both occlusal and buccal microwear patterns, isotopic data, and anatomical adaptations in the paranthropine clade.

## Supporting Information

S1 Table Raw data of all studied variables for all the specimens considered (EXCEL file). (XLSX)

S2 Table Wilks' Lambda test (Rao's approximation) of significance of the differences among groups. (DOCX)

S3 Table One-dimensional ANOVA test of equality of group means. (DOCX)

S4 Table Eigenvalues and percent of total variance explained by the first five discriminant functions derived from the LDA. (DOCX)

S5 Table Pearson correlations between the first five discriminant functions derived from the LDA and the 8 microwear variables considered. (DOCX)

S6 Table Confusion matrix (percentage of post-hoc correctly classified specimens over total group sample) before jack-knife cross-validation. (DOCX)

S7 Table Confusion matrix (percentage of post-hoc correctly classified specimens over total group sample) after jack-knife cross-validation. (DOCX)

S8 Table Similarity matrix between groups derived from the LDA (values are Fisher's distance between pairs of taxa in S1 Table. (DOCX)

S9 Table Significance (P-value) of Fisher's distance (dF) between groups. (DOCX)
