# Supplementary material for: Testing Dietary Hypotheses of East African Hominines Using Buccal Dental Microwear Data
Source: PLoS One. 2016 Nov 16;11(11):e0165447. doi: 10.1371/journal.pone.0165447 (PMC5112956; doi:10.1371/journal.pone.0165447)
Supplement: S2 Table — (DOCX) [file pone.0165447.s002.docx]

**S2 Table.** Wilks' Lambda test (Rao's approximation) of significance of the differences among groups.

| Lambda | 0.133 |
| --- | --- |
| F (observed value) | 3.454 |
| F (critical value) | 1.235 |
| DF1 | 120 |
| DF2 | 1265 |
| *P-*value | < 0.0001 |
| Alfa value | 0.05 |

DF: degrees of freedom. Shaded *P*-values are significant at <5% significance.
