# Supplementary material for: Testing Dietary Hypotheses of East African Hominines Using Buccal Dental Microwear Data
Source: PLoS One. 2016 Nov 16;11(11):e0165447. doi: 10.1371/journal.pone.0165447 (PMC5112956; doi:10.1371/journal.pone.0165447)
Supplement: S3 Table — (DOCX) [file pone.0165447.s003.docx]

**S3 Table.** One-dimensional ANOVA test of equality of group means.

| **Variable** | **Lambda** | **F** | **DF1** | **DF2** | ***P*-value** |
| --- | --- | --- | --- | --- | --- |
| NH | 0.784 | 3.368 | 15 | 183 | < 0.0001 |
| XH | 0.795 | 3.141 | 15 | 183 | 0.000 |
| NV | 0.448 | 15.033 | 15 | 183 | < 0.0001 |
| XV | 0.865 | 1.898 | 15 | 183 | 0.026 |
| NMD | 0.768 | 3.695 | 15 | 183 | < 0.0001 |
| XMD | 0.849 | 2.173 | 15 | 183 | 0.009 |
| NDM | 0.784 | 3.355 | 15 | 183 | < 0.0001 |
| XDM | 0.923 | 1.016 | 15 | 183 | 0.441 |

DF: degrees of freedom. Shaded *P*-values are significant at <5% significance.
