# Supplementary material for: Testing Dietary Hypotheses of East African Hominines Using Buccal Dental Microwear Data
Source: PLoS One. 2016 Nov 16;11(11):e0165447. doi: 10.1371/journal.pone.0165447 (PMC5112956; doi:10.1371/journal.pone.0165447)
Supplement: S4 Table — (DOCX) [file pone.0165447.s004.docx]

**S4 Table.** Eigenvalues and percent of total variance explained by the first five discriminant functions derived from the LDA.

|  | **F1** | **F2** | **F3** | **F4** | **F5** |
| --- | --- | --- | --- | --- | --- |
| Eigenvalue | 1.608 | 0.461 | 0.291 | 0.166 | 0.128 |
| Variance explained (%) | 57.255 | 16.405 | 10.358 | 5.900 | 4.564 |
| % cum. variance | 57.255 | 73.660 | 84.017 | 89.918 | 94.482 |
