# Supplementary material for: Testing Dietary Hypotheses of East African Hominines Using Buccal Dental Microwear Data
Source: PLoS One. 2016 Nov 16;11(11):e0165447. doi: 10.1371/journal.pone.0165447 (PMC5112956; doi:10.1371/journal.pone.0165447)
Supplement: S5 Table — (DOCX) [file pone.0165447.s005.docx]

**S5 Table.** Pearson correlations between the first five discriminant functions derived from the LDA and the 8 microwear variables considered**.**

|  | **F1** | **F2** | **F3** | **F4** | **F5** |
| --- | --- | --- | --- | --- | --- |
| NH | -0.093 | 0.663 | 0.377 | 0.167 | -0.523 |
| XH | -0.279 | -0.359 | 0.579 | 0.447 | 0.001 |
| NV | 0.922 | -0.147 | -0.271 | -0.015 | 0.171 |
| XV | -0.083 | 0.172 | -0.282 | 0.744 | 0.282 |
| NMD | 0.494 | 0.184 | 0.231 | -0.195 | -0.542 |
| XMD | 0.007 | -0.214 | 0.606 | 0.505 | 0.239 |
| NDM | -0.079 | 0.706 | 0.310 | -0.196 | 0.435 |
| XDM | -0.131 | 0.015 | 0.098 | 0.503 | 0.016 |
