# Supplementary material for: Testing Dietary Hypotheses of East African Hominines Using Buccal Dental Microwear Data
Source: PLoS One. 2016 Nov 16;11(11):e0165447. doi: 10.1371/journal.pone.0165447 (PMC5112956; doi:10.1371/journal.pone.0165447)
Supplement: S6 Table — (DOCX) [file pone.0165447.s006.docx]

**S6 Table.** Confusion matrix (percentage of *post-hoc* correctly classified specimens over total group sample) before jack-knife cross-validation.

| **Group** | **Total** | **% Correctly class.** |
| --- | --- | --- |
| *Australopithecus afarensis* | 26 | 65.38% |
| *Australopithecus anamensis* | 5 | 0.00% |
| *Cercocebus torquatus* | 3 | 0.00% |
| *Cercopithecus mitis* | 10 | 40.00% |
| *Chlorocebus pygerythrus* | 15 | 26.67% |
| *Colobus sp.* | 21 | 47.62% |
| *Gorilla beringei graueri* | 7 | 28.57% |
| *Gorilla gorilla gorilla* | 31 | 48.39% |
| *Homo ergaster* | 6 | 16.67% |
| *Homo habilis* | 10 | 60.00% |
| *Mandrillus sphinx* | 4 | 25.00% |
| *Pan troglodytes* | 10 | 10.00% |
| *Papio anubis* | 27 | 85.19% |
| *Paranthropus aethiopicus* | 7 | 14.29% |
| *Paranthropus boisei* | 10 | 50.00% |
| *Theropithecus gelada* | 7 | 42.86% |
| **Total** | 199 | 46.73% |
