# Supplementary material for: Testing Dietary Hypotheses of East African Hominines Using Buccal Dental Microwear Data
Source: PLoS One. 2016 Nov 16;11(11):e0165447. doi: 10.1371/journal.pone.0165447 (PMC5112956; doi:10.1371/journal.pone.0165447)
Supplement: S8 Table — (DOCX) [file pone.0165447.s008.docx]

**S8 Table.** Similarity matrix between groups derived from the LDA (values are Fisher's distance between pairs of taxa in S1 Table.

|  | ***A. afarensis*** | ***A. anamensis*** | ***C. torquatus*** | ***C. mitis*** | ***Ch. pygerythrus*** | ***Colobus sp.*** | ***G. beringei graueri*** | ***G. g. gorilla*** | ***H. ergaster*** | ***H.o habilis*** | ***M. sphinx*** | ***P. troglodytes*** | ***Papio anubis*** | ***P. aethiopicus*** | ***P. boisei*** | ***Th. gelada*** |
| --- | --- | --- | --- | --- | --- | --- | --- | --- | --- | --- | --- | --- | --- | --- | --- | --- |
| *Australopithecus afarensis* |  |  |  |  |  |  |  |  |  |  |  |  |  |  |  |  |
| *Australopithecus anamensis* | 2.552 |  |  |  |  |  |  |  |  |  |  |  |  |  |  |  |
| *Cercocebus torquatus* | 2.585 | 1.566 |  |  |  |  |  |  |  |  |  |  |  |  |  |  |
| *Cercopithecus mitis* | 10.166 | 1.255 | 2.350 |  |  |  |  |  |  |  |  |  |  |  |  |  |
| *Chlorocebus pygerythrus* | 7.975 | 0.788 | 1.394 | 1.123 |  |  |  |  |  |  |  |  |  |  |  |  |
| *Colobus sp.* | 3.910 | 2.785 | 2.729 | 9.306 | 6.113 |  |  |  |  |  |  |  |  |  |  |  |
| *Gorilla beringei graueri* | 2.939 | 2.320 | 1.936 | 5.073 | 3.018 | 2.207 |  |  |  |  |  |  |  |  |  |  |
| *Gorilla gorilla gorilla* | 5.834 | 1.993 | 1.158 | 5.888 | 2.443 | 3.688 | 1.121 |  |  |  |  |  |  |  |  |  |
| *Homo ergaster* | 2.003 | 1.533 | 1.225 | 4.627 | 2.883 | 1.334 | 1.958 | 2.045 |  |  |  |  |  |  |  |  |
| *Homo habilis* | 3.618 | 4.279 | 3.689 | 11.425 | 9.525 | 3.439 | 3.881 | 7.345 | 2.775 |  |  |  |  |  |  |  |
| *Mandrillus sphinx* | 2.409 | 1.033 | 0.545 | 1.378 | 0.681 | 3.072 | 1.547 | 0.955 | 1.537 | 4.606 |  |  |  |  |  |  |
| *Pan troglodytes* | 2.290 | 2.265 | 1.721 | 5.748 | 3.189 | 1.154 | 0.625 | 0.859 | 1.266 | 4.388 | 1.531 |  |  |  |  |  |
| *Papio anubis* | 11.492 | 0.562 | 3.635 | 2.474 | 2.486 | 9.530 | 4.351 | 8.479 | 4.653 | 11.199 | 2.394 | 5.706 |  |  |  |  |
| *Paranthropus aethiopicus* | 2.998 | 4.330 | 3.878 | 10.916 | 8.267 | 1.997 | 2.364 | 5.068 | 2.755 | 1.067 | 4.714 | 2.591 | 8.998 |  |  |  |
| *Paranthropus boisei* | 4.381 | 3.913 | 3.791 | 11.817 | 8.939 | 1.985 | 2.884 | 6.173 | 2.272 | 1.794 | 5.004 | 3.030 | 9.966 | 0.431 |  |  |
| *Theropithecus gelada* | 4.712 | 1.192 | 1.864 | 2.485 | 0.994 | 2.829 | 1.802 | 1.548 | 2.501 | 4.748 | 1.287 | 2.334 | 1.938 | 4.470 | 4.610 |  |
