# Supplementary material for: Testing Dietary Hypotheses of East African Hominines Using Buccal Dental Microwear Data
Source: PLoS One. 2016 Nov 16;11(11):e0165447. doi: 10.1371/journal.pone.0165447 (PMC5112956; doi:10.1371/journal.pone.0165447)
Supplement: S9 Table — (DOCX) [file pone.0165447.s009.docx]

**S9 Table.** Significance (*P*-value) of Fisher's distance (d_F_) between groups.

|  | ***A. afarensis*** | ***A. anamensis*** | ***C. torquatus*** | ***C. mitis*** | ***Ch. pygerythrus*** | ***Colobus sp.*** | ***G. beringei graueri*** | ***G. g. gorilla*** | ***H. ergaster*** | ***H.o habilis*** | ***M. sphinx*** | ***P. troglodytes*** | ***Papio anubis*** | ***P. aethiopicus*** | ***P. boisei*** | ***Th. gelada*** |
| --- | --- | --- | --- | --- | --- | --- | --- | --- | --- | --- | --- | --- | --- | --- | --- | --- |
| *A. afarensis* |  |  |  |  |  |  |  |  |  |  |  |  |  |  |  |  |
| *A. anamensis* | 0.012 |  |  |  |  |  |  |  |  |  |  |  |  |  |  |  |
| *C. torquatus* | 0.011 | 0.138 |  |  |  |  |  |  |  |  |  |  |  |  |  |  |
| *C. mitis* | < 0.0001 | 0.270 | 0.020 |  |  |  |  |  |  |  |  |  |  |  |  |  |
| *Ch. pygerythrus* | < 0.0001 | 0.614 | 0.202 | 0.350 |  |  |  |  |  |  |  |  |  |  |  |  |
| *Colobus sp.* | 0.000 | 0.006 | 0.007 | < 0.0001 | < 0.0001 |  |  |  |  |  |  |  |  |  |  |  |
| *G. beringei graueri* | 0.004 | 0.022 | 0.057 | < 0.0001 | 0.003 | 0.029 |  |  |  |  |  |  |  |  |  |  |
| *G. gorilla gorilla* | < 0.0001 | 0.050 | 0.327 | < 0.0001 | 0.016 | 0.001 | 0.351 |  |  |  |  |  |  |  |  |  |
| *H. ergaster* | 0.049 | 0.149 | 0.287 | < 0.0001 | 0.005 | 0.229 | 0.054 | 0.044 |  |  |  |  |  |  |  |  |
| *H. habilis* | 0.001 | 0.000 | 0.001 | < 0.0001 | < 0.0001 | 0.001 | 0.000 | < 0.0001 | 0.006 |  |  |  |  |  |  |  |
| *Mandrillus sphinx* | 0.017 | 0.413 | 0.821 | 0.209 | 0.707 | 0.003 | 0.144 | 0.473 | 0.147 | < 0.0001 |  |  |  |  |  |  |
| *Pan troglodytes* | 0.023 | 0.025 | 0.096 | < 0.0001 | 0.002 | 0.330 | 0.756 | 0.552 | 0.264 | < 0.0001 | 0.149 |  |  |  |  |  |
| *Papio anubis* | < 0.0001 | 0.808 | 0.001 | 0.014 | 0.014 | < 0.0001 | < 0.0001 | < 0.0001 | < 0.0001 | < 0.0001 | 0.018 | < 0.0001 |  |  |  |  |
| *P. aethiopicus* | 0.004 | < 0.0001 | 0.000 | < 0.0001 | < 0.0001 | 0.049 | 0.019 | < 0.0001 | 0.007 | 0.388 | < 0.0001 | 0.011 | < 0.0001 |  |  |  |
| *P. boisei* | < 0.0001 | 0.000 | 0.000 | < 0.0001 | < 0.0001 | 0.051 | 0.005 | < 0.0001 | 0.025 | 0.081 | < 0.0001 | 0.003 | < 0.0001 | 0.902 |  |  |
| *Th. gelada* | < 0.0001 | 0.307 | 0.068 | 0.014 | 0.442 | 0.006 | 0.079 | 0.144 | 0.013 | < 0.0001 | 0.253 | 0.021 | 0.057 | < 0.0001 | < 0.0001 |  |
